# Development of a video camera-type kayak motion capture system to measure water kayaking

**DOI:** 10.7717/peerj.15227

**Published:** 2023-07-21

**Authors:** Shigeaki Miyazaki, Go Yamako, Ryo Kimura, Niroshan G. Punchihewa, Tsubasa Kawaguchi, Hideki Arakawa, Etsuo Chosa

**Affiliations:** 1 Rehabilitation Unit, University of Miyazaki Hospital, Miyazaki, Japan; 2 Department of Mechanical Engineering, Faculty of Engineering, University of Miyazaki, Miyazaki, Japan; 3 Department of Orthopaedic Surgery, Faculty of Medicine, University of Miyazaki, Miyazaki, Japan

**Keywords:** Kayak, Biomechanics, Motion capture system, Accuracy, Low-back injury

## Abstract

**Background:**

In kayaking, trunk motion is one of the important factors that prevent injury and improve performance. Kinematic studies in kayaking have been reported in laboratory settings using paddling simulators and ergometers. However, such studies do not reflect kayaking on water, the actual competitive environment. Therefore, we developed a video camera-type kayak motion capture system (KMCS) wherein action cameras were fixed to a kayak to capture images of markers attached to an athlete’s body. This study aimed to compare the kinematic data between KMCS and an optical motion capture system (OMCS) in kayaking and to determine the accuracy of the KMCS analysis.

**Methods:**

In a competition, five elite junior female kayak athletes performed kayak paddling under the unloaded condition using a kayak. The kayak was secured using a tri-folding bench and a towel, and twenty strokes were recorded during maximal paddling. One stroke was defined as the period from right catch to left catch, and the first six strokes were used to evaluate the accuracy. Trunk angles (tilting, turning, and rotation) were examined with the simultaneous use of KMCS and OMCS, and the differences between these systems were evaluated. To ensure reliability, intraclass correlation coefficient (ICC; a two-way mixed model for absolute agreement) was calculated for each angle. Furthermore, Bland–Altman analysis was performed to understand the agreement between the two systems.

**Results:**

Root mean square errors (RMSEs) were 1.42° and 3.94° for turning and rotation, respectively, and mean absolute errors (MAEs) were 1.08° and 3.00° for turning and rotation, respectively. The RMSE and MAE for tilting were 2.43° and 1.76°, respectively, which indicated that the validity was comparable to that of other angles. However, the range of motion in tilting was lower than that in turning and rotation. Bland–Altman analysis showed good agreement in the total range of motion, with mean bias values of −0.84°, −0.07°, and −0.41° for tilting, turning, and rotation, respectively. The ICCs for tilting, turning, and rotation were 0.966, 0.985, and 0.973, respectively, and showed excellent reliability.

**Conclusions:**

The newly developed KMCS effectively measured the trunk motion with good accuracy in kayaking. In future studies, we intend to use KMCS to measure kayaking on water and collect data for performance improvement and injury prevention.

## Introduction

In canoeing, there are two types of boats, namely, kayaks and canoes. In a kayak, the paddler is seated and uses a double-bladed paddle, whereas in a canoe, the paddler kneels and uses a single-bladed paddle to propel the boat forward (International Canoe Federation, 2022b). A canoe sprint is one of the disciplines in canoeing. The sprint takes place on a flatwater course, and races are contested using canoes and kayaks (International Canoe Federation, 2022a). Kayaking is achieved by transferring kinetic energy not only to the paddle but also to other points of contact of the paddler with the kayak (footrest and seat). In kayaking, four stroke positions are identified, namely, catch, immersion, extraction, and release. Catch is defined as the first contact between the paddle blade and water; immersion is when the blade is fully submerged; extraction is the last instance of full-blade submersion; and release is the last contact between the blade and water. Based on these positions, the stroke motion in kayaking is categorized into two phases: water and aerial (Gomes et al., 2022; Liu et al., 2021; Kong, Tay & Pan, 2020; Tay & Kong, 2018; McDonnell, Hume & Nolte, 2012).

Kameyama et al. (1999) reported that the most common site of sports injuries among canoeists was the lumbar region, followed by the shoulder and hand joints. The researchers also observed that low-back injury was the most common complication among kayakers. In a survey of Hawaiian outrigger canoeists, shoulders (40%), back (26%), and hands/wrists (10%) accounted for the majority of musculoskeletal injuries incurred in paddling sports (Haley & Nichols, 2009). Griffin et al. (2020) identified and compared the rates and types of injuries sustained by 583 kayak and/or ocean surf ski race competitors. The investigators found that paddling-related injuries mainly occurred in the shoulder (31%), lower back (23.5%), wrist (16.5%), neck (13.7%), and elbow (11.0%). These reports indicate that shoulder and lower back injuries are the most common musculoskeletal injuries in kayakers. In a study by Kameyama et al. (1999), 25% of the 417 respondents complained of concurrent shoulder and low-back pain. The pathologies included spondylolysis (17.5%), myofascial pain (15.9%), spondylosis deformations (12.7%), and disc herniation (3.2%). Abraham & Stepkovitch (2012) reported that nearly one-third of the competitors with shoulder injury had concurrent thoracic or lumbar spine complaints. The authors reasoned that kyphosis (flexed posture) of the thoracic spine may cause biomechanical issues, such as reduced upward scapular rotation, muscle fatigue, and altered shoulder girdle muscle activation patterns. They also reported that these problems may contribute to shoulder impingement syndrome and glenohumeral joint instability. Therefore, kayaking while the thoracic spine is in kyphosis (flexed posture) or in a posterior pelvic tilt should be avoided not only from a performance standpoint but also from the perspective of preventing injury.

One method of demonstrating the causes of shoulder and low-back injury is motion analysis using a three-dimensional (3D) motion analyzer. An optical motion capture system (OMCS) is the gold standard for kinematic joint analysis owing to its simplicity and accuracy (Bertozzi et al., 2022; Bjerkefors et al., 2019, 2018). Kinematic studies in kayaking have been performed with the use of paddling simulators (Limonta et al., 2010) and ergometers (Bertozzi et al., 2022; Klitgaard et al., 2021; Bjerkefors et al., 2019, 2018; Fleming et al., 2012; Begon, Colloud & Sardain, 2010). However, these methods do not reflect the competitive environment in kayaking because motion is evaluated in laboratory settings. OMCS cannot be used when kayaking is performed in its ecological condition, *i.e*., on water, owing to measurement space restrictions and environmental factors. Klitgaard et al. (2021) compared kayaking on an ergometer with that on water using an inertial motion capture system (IMCS) and revealed differences in kayak motion. This research emphasizes the importance of on-water kinematic analysis in kayaking. Therefore, we developed a video camera-type kayak motion capture system (KMCS) wherein action cameras were fixed to a kayak to capture images of markers attached to an athlete’s body. In the future, we plan to use this system to analyze kayaking characteristics and clarify the cause of low-back injury. As a preliminary step, the validity of the 3D measurement data should be proven. Therefore, this study aimed to compare kinematic data between KMCS and OMCS in kayaking and to determine the accuracy of KMCS analysis.

## Materials and Methods

### Study design and ethical statement

This study was conducted after receiving the approval of the Research Ethics Committee of the Faculty of Medicine, University of Miyazaki (Approval number 2013-027 (O) and O-0448). As the participants were minors, the study purpose and content were explained orally and in a written form to the participants and their parents, and written informed consent was obtained.

### Participants

The participants included in this study were five healthy female students (age: 17.0 ± 0.0 years (mean ± standard deviation (SD)); height, 1.60 ± 0.03 m; weight, 56.4 ± 6.9 kg) who were elite junior kayak athletes without pain induced by a kayaking motion. None of the participants were injured or recovering from an injury at the time of testing.

### Procedures

**Figure 1:**
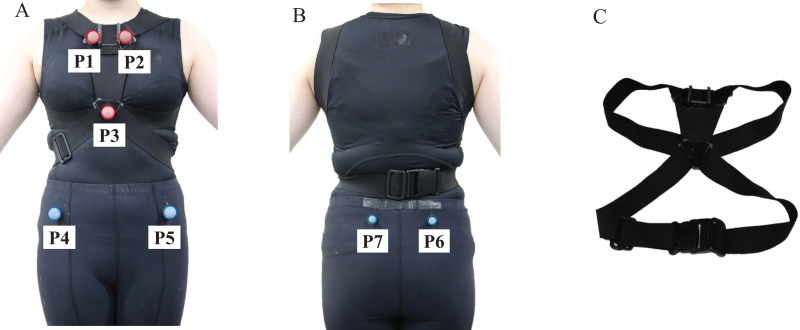
(A–C) Marker set.

**Figure 2:**
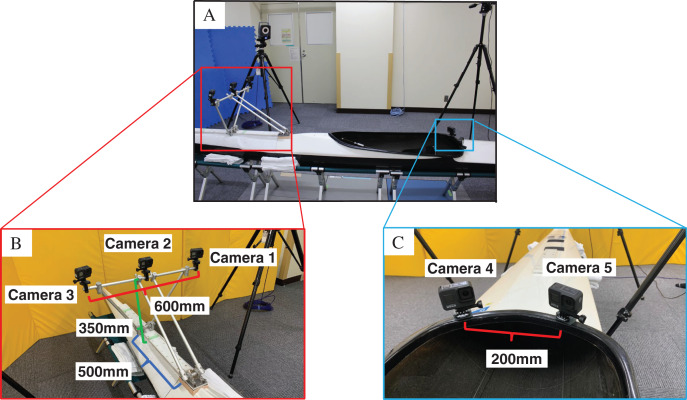
(A–C) Video camera type kayak motion capture system (KMCS).

Participants wore clothing that adhered to their skin (Under Armour, Baltimore, MD, USA). Reflective markers were attached to three sites on the thorax and four on the pelvis. The thoracic markers P1–P3 were placed on the right and left sternoclavicular joints and the xiphoid process, and the pelvic markers P4–P7 were placed on the right and left superior anterior iliac spines and the right and left superior posterior iliac spines (Fig. 1A). The spherical markers were 20 mm in diameter for the front five (P1–P5) and 10 mm in diameter for the rear two (P6, P7). As the front camera was installed at a depth of 500 mm, which is farther than the rear camera, the size of the front marker was increased to ensure its visibility (Figs. 1A, 1B, 2A and 2B). Furthermore, a marker band with a marker was prepared to suppress the deviation between the marker and the body surface to the best possible extent. The marker band was secured to the participant’s body using an elastic band. Acrylonitrile butadiene styrene (ABS) resin plates were used for the markers (Fig. 1C).

Kayaking motion was measured in our laboratory using a kayak meant for competition use and paddles. The kayak was fixed using four tri-folding benches (Coleman Inc., Wichita, KS, USA) and towels. A stroke cycle was defined as previously described by Tay & Kong (2018). In sprint kayaking, a complete stroke cycle beginning and ending on one side (*e.g*., from the right catch to the next right catch) is commonly considered as two strokes. The initial position was considered as the right catch, which was defined as the moment of maximal left trunk rotation (−direction), whereas the left catch was defined as the moment of maximal right trunk rotation (+direction). Twenty strokes during maximal paddling (top speed) were recorded twice for each participant. The top speed was defined as the time when the paddling frequency reached its maximum. The participants warmed-up and practiced so that they could kayak naturally. Moreover, they adjusted the position of the footrest and seat according to their preference. Before testing, they completed a preparation routine, including stretching and warm-up kayaking. Instructions to quantify kayaking included the following: (1) to kayak as if on water; (2) to perform at the maximum level; and (3) to take 5 min of rest between the measurements.

### KMCS

In this study, we developed a KMCS that measures trunk motion by installing five action cameras (Go Pro HERO8 Black; Go Pro Inc., San Mateo, CA, USA) on the kayak and using spherical markers attached to the participants’ bodies. The camera was set to a frame rate of 120 fps, an image size of 1,920 × 1,080 pixels, a narrow-angle forward camera, and a wide-angle rear camera. Additionally, the ISO settings were set to a minimum of 100 and a maximum of 1,600. Motion analysis was performed with KMCS using the following procedures: (1) photographing, (2) calculation of 3D marker coordinates, and (3) calculation of the trunk angle.

#### Photographic equipment

The action camera was fixed securely using aluminum frames and ABS resin jig. Three cameras were mounted at the front of the kayak, and two cameras were placed at the rear (Fig. 2). The camera angle was adjusted to capture all markers. The mass of the action camera was 147 g, and the total mass of the fixation jig was 1.2 kg. The distance between the three cameras at the front was measured. In case of KMCS, the direct linear transformation (DLT) method (Abdel-Aziz & Karara, 2015) was used to calculate the 3D marker coordinates. Therefore, preliminary experiments were conducted to determine the distance between the cameras. The error of the distance between the markers was compared when the distance was changed. Table 1 shows the results. Although the error of Z-axis coordinates (out-of-plane direction) was the smallest, with a camera distance of 800 mm, the jig used to fix the camera should be small and lightweight to minimize any impact while kayaking. A camera distance of 600 mm was almost similar in error as that of 800 mm. Therefore, a camera distance of 600 mm was adopted in this study. The higher the front camera position, the easier it is for pelvic markers P4 and P5 to capture the image, thus improving data accuracy. However, higher the camera position greater is the induced camera sway. The sitting height of the participants was measured to determine the lowest height at which the five anterior markers (P1–P5) could be captured. A height of 350 mm was used in this study (Fig. 2B).

**Table 1:** Error in distance between markers when distance between cameras is varied.

Distance between cameras	X axis direction	Y axis direction	Z axis direction
200 (mm)	0.66	0.59	3.03
400 (mm)	0.43	0.48	2.18
600 (mm)	0.23	0.28	1.26
800 (mm)	0.32	0.28	1.11

#### Action camera distortion correction

A checkerboard (550 mm × 600 mm) was photographed with each camera to correct camera lens distortion. Distortion correction values ( ${k_1}$, ${k_2}$) were calculated using MATLAB (MathWorks Inc., Natick, MA, USA) with Eq. (1).

(1) $$\left. {\matrix{ {{u_d} = u\left( {1 + {k_1}{r^2} + {k_2}{r^4}} \right)} \cr {{v_d} = v\left( {1 + {k_1}{r^2} + {k_2}{r^4}} \right)} \cr } } \right\}$$

Image coordinates were obtained by photographing a point in a 3D space using an ideal lens without distortion ( $u,v$) and a lens with distortion ( ${u_d},{v_d}$); *r* denotes the distance from the image center.

#### Calculation of marker 3D coordinates

**Figure 3:**
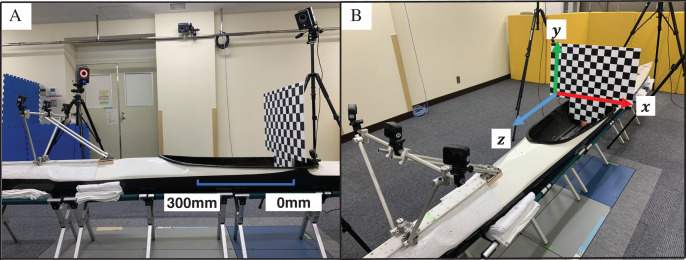
(A–C) Calculation of marker 3D coordinates.

The 3D coordinates of each marker were calculated using the DLT method (Abdel-Aziz & Karara, 2015) with multiple cameras to obtain the image coordinates of the calibration points ( $u$, $v$) by photographing those points (control points) that represent the known real-space coordinates ( $x$, $y$, $z$) with each camera in advance. From this relationship between ( $x$, $y$, $z$) and ( $u$, $v$), camera parameters were calculated to convert the image coordinates to 3D coordinates. The 3D coordinates of the marker were calculated using the camera parameters and image coordinates of the marker. Initially, 11 camera parameters ( ${L_1}$– ${L_{11}}$) were obtained. The checkerboard (size: 550 mm × 600 mm, intersection spacing: 50 mm) was moved at 25-mm intervals between 0 and 300 mm behind the cockpit and photographed at 13 locations (Fig. 3A). Using image coordinates ( ${u_i}$, ${v_i}$) and real-space coordinates ( ${x_i}$, ${y_i}$, ${z_i}$) of each calibration point (intersection of checkerboards), camera parameters were calculated with the least-squares method based on Eq. (2). The control points of front and rear cameras were 1,781 and 260 points, respectively.

(2a) $${u_i} = \displaystyle{{{L_1}{x_i} + {L_2}{y_i} + {L_3}{z_i} + {L_4}} \over {{L_9}{x_i} + {L_{10}}{y_i} + {L_{11}}{z_i} + 1}}$$

(2b) $${v_i} = \displaystyle{{{L_5}{x_i} + {L_6}{y_i} + {L_7}{z_i} + {L_8}} \over {{L_9}{x_i} + {L_{10}}{y_i} + {L_{11}}{z_i} + 1}}$$

In the global coordinate system, the point of origin was set at the rear part of the cockpit, with the right direction defined as the X-axis and the upward direction as the Y-axis. The Z-axis was the outer product of the X-axis and Y-axis, and the forward direction of the kayak was assumed to be positive (Fig. 3B). The image coordinates of the markers captured with each camera were obtained semi-automatically by combining a template matching technique and circle detection using the Hough transform. When automatic detection *via* template matching was difficult (when the correlation coefficient was <0.6), the image coordinates of the marker were recorded manually. The 3D coordinates ( $x$, $y$, $z$) of each marker were calculated using Eq. (3) based on camera parameters and image coordinates of the marker.

(3) $$\left[ {\matrix{ {L_4^1 - {u^1}} \cr {L_8^1 - {v^1}} \cr {L_4^2 - {u^2}} \cr {L_8^2 - {v^2}} \cr \vdots \cr {L_4^n - {u^n}} \cr {L_8^n - {v^n}} \cr } } \right] = \left[ {\matrix{ {L_9^1{u^1} - L_1^1} & {L_{10}^1{u^1} - L_2^1} & {L_{11}^1{u^1} - L_3^1} \cr {L_9^1{v^1} - L_5^1} & {L_{10}^1{v^1} - L_6^1} & {L_{11}^1{v^1} - L_7^1} \cr {L_9^2{u^2} - L_1^2} & {L_{10}^2{u^2} - L_2^2} & {L_{11}^2{u^2} - L_3^2} \cr {L_9^2{v^2} - L_5^2} & {L_{10}^2{v^2} - L_6^2} & {L_{11}^2{v^2} - L_7^2} \cr {} & \vdots & {} \cr {L_9^n{u^n} - L_1^n} & {L_{10}^n{u^n} - L_2^n} & {L_{11}^n{u^n} - L_3^n} \cr {L_9^n{v^n} - L_5^n} & {L_{10}^n{v^n} - L_6^n} & {L_{11}^n{v^n} - L_7^n} \cr } } \right]\left[ {\matrix{ x \cr y \cr z \cr } } \right]$$

where, $L_1^n - L_{11}^n\;$ are the camera parameters of camera n and ( ${u^n},{v^n}$) are image coordinates of the marker obtained with camera n. Furthermore, if the marker was blocked by paddles, arms, *etc*., or if the 3D coordinates could not be calculated using the camera, defect points were interpolated using cubic spline interpolation.

#### Calculation of the trunk angle

**Figure 4:**
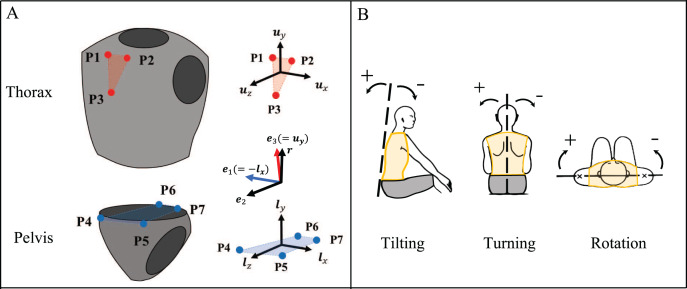
(A, B) Calculation of the trunk angle.

To determine the trunk angle, tilting, turning, and rotation were defined using the Joint Coordinate System recommended by the International Society of Biomechanics (Wu et al., 2002, 2005; Cole et al., 1993). To start with, the local coordinate system of the thorax was constructed using the positions of the marker ${{\rm P}_1},{{\rm P}_3}$ (Fig. 4A). Each unit base vector of the thorax coordinate system was set to ${{ u}_x},{ \; }{{ u}_y},{ \; }{{ u}_z}$ and was calculated using Eq. (4).

(4a) $${{ u}_x} = \displaystyle{{{{ P}_2} - {{ P}_1}} \over {\left| {{{ P}_2} - {{ P}_1}} \right|}}$$

(4b) $${{ u}_z} = \displaystyle{{\left( {{{ P}_3} - {{ P}_1}} \right) \times {{ u}_x}} \over {\left| {\left( {{{ P}_3} - {{ P}_1}} \right) \times {{ u}_x}} \right|}}$$

(4c) $${{ u}_y} = {{ u}_z} \times {{ u}_x}$$

The local coordinate system of the pelvis was then created using the positions of ${\rm \; }{{ P}_4} - {{ P}_7}$. The respective unit base vectors of the pelvic coordinate system were defined as ${{ l}_x},\; {{ l}_y},\; {{ l}_z}$ using Eq. (5).

(5a) $${{ l}_x} = \displaystyle{{\left( {{{ P}_5} + {{ P}_7}} \right) - \left( {{{ P}_4} + {{ P}_6}} \right)} \over {\left| {\left( {{{ P}_5} + {{ P}_7}} \right) - \left( {{{ P}_4} + {{ P}_6}} \right)} \right|}}$$

(5b) $${{ l}_y} = \displaystyle{{\left\{ {\left( {{{ P}_4} + {{ P}_5}} \right) - \left( {{{ P}_6} + {{ P}_7}} \right)} \right\} \times {{ l}_x}} \over {\left| {\left\{ {\left( {{{ P}_4} + {{ P}_5}} \right) - \left( {{{ P}_6} + {{ P}_7}} \right)} \right\} \times {{ l}_x}} \right|}}$$

(5c) $${{ l}_z} = {{ l}_x} \times {{ l}_y}$$

Trunk angles (tilting, turning, and rotation) were calculated from the unit vectors of the thorax and pelvis coordinate systems using the Joint Coordinate System of spine (Punchihewa et al., 2020). The floating axis of ${{ e}_2}$ was calculated with Eq. (6) using $- {{ l}_x}\left( { = {{ e}_1}} \right)$ of the pelvis and ${{ u}_y}\left( { = {{ e}_3}} \right)$ of the thorax.

(6) $${{ e}_2} = \displaystyle{{{{ e}_3} \times {{ e}_1}} \over {\left| {{{ e}_3} \times {{ e}_1}} \right|}}$$

The ${{ e}_1}$ of the pelvis was considered as the flexion axis, whereas the ${ \; }{{ e}_3}{ \; }$of the thorax was considered as the rotation axis of the spine. Trunk angles were calculated using Eq. (7) (Fig. 4B).

(7a) $${\rm Tilting} = {\cos ^{ - 1}}\left( {{{ e}_2}*{{ l}_z}} \right)*sgn\left( {{{ e}_2}*{{ l}_y}} \right)$$

(7b) $${\rm Turning} = {\cos ^{ - 1}}\left( {{ r}*{{ e}_3}} \right)*sgn\left( {{{ e}_3}*{{ l}_x}} \right)\; \;$$

(7c) $$Rotation = {\cos ^{ - 1}}\left( {{{ e}_2}*{{ u}_z}} \right)*sgn\left( {{{ e}_2}* - {{ u}_x}} \right)$$

(7d) $${ r} = \displaystyle{{{{ e}_1} \times {{ e}_2}} \over {\left| {{{ e}_1} \times {{ e}_2}} \right|}}$$

After calculating the trunk angles, filtering was performed using a second-order low-pass Butterworth filter at a cutoff frequency of 3 Hz.

### OMCS

**Figure 5:**
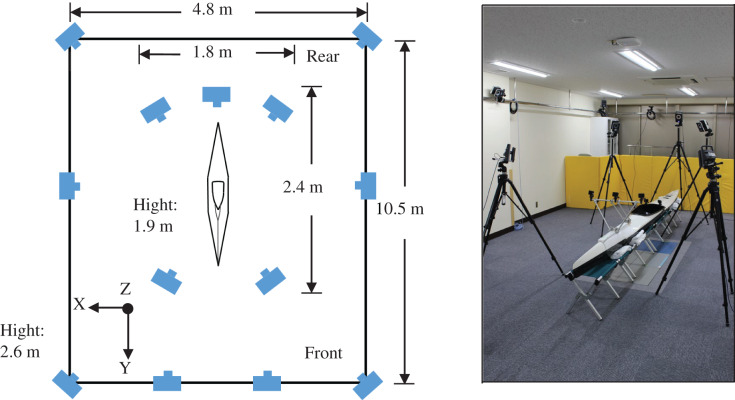
Optical motion capture system (OMCS).

Thirteen infrared cameras (120 Hz; MX T20-S and Vantage 8; Vicon Motion Systems, London, UK) were used for OMCS. Infrared cameras were installed in areas where all markers could be measured (Fig. 5). After installing the cameras, the camera mask and calibration were performed. The calibration was set at a reprojection error of <0.2 mm for each camera. To synchronize OMCS and KMCS, the LED light and 5V voltage were used as triggers. In the global coordinate system, the point of origin was set approximately at the center of the measurement area. The forward direction of the kayak was the Y-axis, and the right direction was defined as the X-axis, which was orthogonal to the Y-axis. Furthermore, the Z-axis was vertically oriented upward (Fig. 5).

### Statistical analysis

#### Laboratory measurements

Trunk angles were calculated using one of the two trials of kayaking, and the first six strokes of maximal paddling were analyzed for measurement accuracy. To analyze the motion of the best performance, data from the motion that the participants perceived to be their best were used as representative values. The accuracy was evaluated based on the root mean square error (RMSE) and mean absolute error (MAE) between the OMCS and KMCS measurement data. An RMSE of <5° was considered excellent and that between 5° and 10° as good (Koo & Li, 2016).

Data were collected as previously described by Punchihewa et al. (2020). To evaluate reliability, intraclass correlation coefficient (ICC; a two-way mixed model for absolute agreement) was calculated using the Statistical Package for the Social Sciences software (version 28.0, released 2021; IBM Corp., Armonk, NY, USA). An ICC of <0.5 was considered poor, 0.5–0.75 as moderate, 0.75–0.9 as good, and >0.9 as excellent (Pasciuto et al., 2015). Bland–Altman analysis was performed to understand the agreement between the two measurement systems (Altman & Bland, 1983). The upper and lower limits were calculated as mean bias ±1.96 times the SD.

## Results

### Kayaking description using KMCS

**Figure 6:**
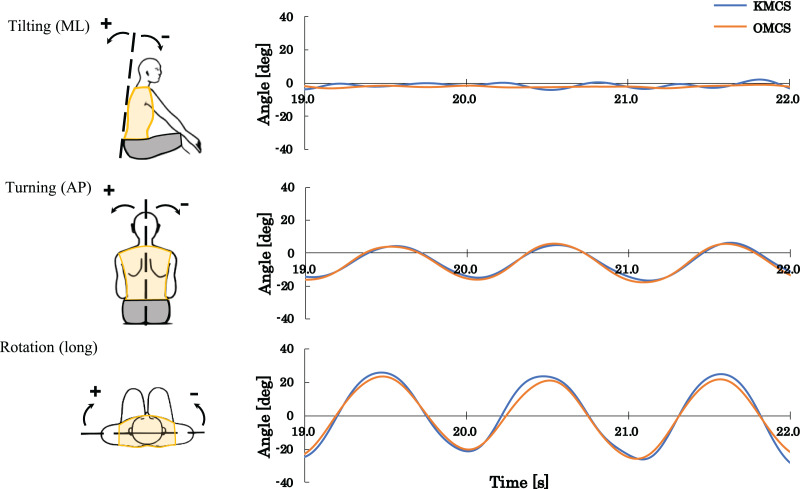
Typical graph of time-dependent change in angular displacement of the trunk measured using the video camera-type kayak motion capture system (KMCS) and the optical motion capture system (OMCS).

The time-dependent change in angular displacement of the trunk in kayaking was as follows: for the right stroke (right catch to left catch), there was a shift in turning and rotation from negative to positive. Conversely, for the left stroke (left catch to right catch), turning and rotation changed from positive to negative. There was minimal change in tilt (Fig. 6).

### Validity and reliability of KMCS

When kayaking kinematics were measured, turning and rotation showed excellent validity (Table 2). Both RMSEs and MAEs were <5° and deemed accurate (Koo & Li, 2016). For tilting, the RMSE and MAE were comparable to those of other angles; however, the ROM was relatively small (Fig. 6).

**Table 2:** RMSE and MAE were reported as angular displacements of the respective trunk kinematics.

Segment	Axis	RMSE (°) (angular displacement)	MAE (°) (angular displacement)
Trunk	Tilting (ML)	2.43	1.76
	Turning (AP)	1.42	1.08
	Rotation (long)	3.94	3.00

**Note:**

RMSE, root mean squared error; MAE, mean absolute error; ML, medio-lateral axis; AP, antero-posterior axis; long, longitudinal axis.

Bland–Altman analysis showed good agreement in angular displacements of total range of motion, with mean bias values of −0.84°, −0.07°, and −0.41° for tilting, turning, and rotation, respectively. The ICCs showed excellent reliabilities of 0.966, 0.985, and 0.973 for tilting, turning, and rotation, respectively (Table 3).

**Table 3:** Mean (SD) measurements of KMCS and OMCS, mean bias with UB and LB of the LOA, and ICC between measurement systems in angular displacement of the trunk.

Segment	Axis	Angular displacement (°)				
		Total range of motion KMCS Mean (SD)	Total range of motion OMCS Mean (SD)	Bias	LOA (UB, LB)	ICC
Trunk	Tilting (ML)	9.92 (3.13)	7.28 (4.28)	−0.84	(3.63, −5.32)	0.966
	Turning (AP)	24.32 (7.43)	24.55 (7.96)	−0.07	(2.71, −2.84)	0.985
	Rotation (long)	52.89 (11.16)	47.68 (11.60)	−0.41	(7.28, −8.10)	0.973

**Note:**

KMCS, video camera type kayak motion capture system; OMCS, optical motion capture system; SD, standard deviation; LOA, limits of agreement; UB, upper bound; LB, lower bound; ICC, intra-class correlation coefficient; ML, medio-lateral axis; AP, antero-posterior axis; long, longitudinal axis.

## Discussion

The most important finding of this study was that the RMSE and MAE were <5° for tilting, turning, and rotation. The Bland–Altman analysis showed good agreement, and all ICCs exhibited excellent reliability. These results suggest that KMCS measured the trunk motion effectively, with good accuracy in kayaking. Tilting demonstrated the same validity as that of other angles, but the ROM was relatively small. Therefore, detecting slight movements in tilt during kayaking may be difficult (Bertozzi et al., 2022; Bjerkefors et al., 2018). The tilting depends on the accuracy of the Z-axis of the 3D coordinates. Although a greater distance between cameras is expected to improve the Z-axis accuracy, the current system does not permit it. Improper trunk motion would induce low-back and shoulder injuries (Abraham & Stepkovitch, 2012; Kameyama et al., 1999). Limonta et al. (2010) found that trunk motion, including that of the pelvis, was an important factor for effective kayaking. To the best of our knowledge, no previous studies have used OMCS to analyze kayaking on water. In this study, the trunk angle was compared between KMCS and OMCS in kayaking, and the accuracy of KMCS analysis was observed to be good.

Klitgaard et al. (2021) compared the performance on the ergometer and that on water and noted differences in thoracolumbar joint, elbow, shoulder, and knee kinematics. The researchers further reported that the number of strokes on the ergometer (122.1 ± 6.8 strokes/min) was significantly higher than that on water (107.1 ± 4.6 strokes/min). These results indicate that the kayak ergometer may not replicate on-water kinematics. Therefore, future analyses should focus on kayaking on water to prevent injury and improve the performance.

The advantage of KMCS is its ability to classify kayaking into two phases (water and aerial) for motion analysis (McDonnell, Hume & Nolte, 2012). Kayaking involves actions directly connected to athletic ability and injury; thus, behaviors should be converted to a numeric form and quantified for performance improvement and injury prevention. The motion mechanism can be interpreted from continuous body motion changes by classifying kayaking into two phases and analyzing the motions involved, which is lacking in IMCS. In KMCS, the trunk motion in kayaking can be confirmed using camera images, which allows the athlete to visually grasp “what his/her actions are going to be” and provides feedback. Additionally, IMCS causes data drift when continuous measurements are obtained for long periods of time. The disadvantages of the KMCS are that the camera only can be measured using a dedicated kayak because it is installed in the kayak and the weight of the camera and jig is added.

This study has certain limitations. First, the position at which the camera can be installed in the kayak is restricted. Although increasing the distance between the cameras might improve the accuracy in tilting, it is difficult to increase the distance between the cameras further because the weight of the fixation jig cannot be reduced further and the space to fix it is limited. Second, this system was evaluated indoors in our laboratory. Further evaluation outdoors and in different ambient conditions is required to minimize intra-rater variability and to ensure that the results are reproducible. Third, kayaking was measured while unloaded, which might greatly affect the kinematics. Hence, we intend to examine the accuracy of the analysis using a Dansprint kayak ergometer (Dansprint Balance, Dansprint, Hvidovre, Denmark) with a balance board that simulates resistance. In the future, the advantages of KMCS, which can perform motion analysis by classifying it into phases, combined with the advantages of IMCS, which can analyze shoulder and elbow kinematics, are expected to aid in determining the factors causing shoulder and lower back injuries.

## Conclusions

We developed an action camera-based motion capture system to evaluate on-water kayaking. Kinematic data from KMCS and OMCS in kayaking were compared to evaluate the accuracy of KMCS analysis. Based on the findings, KMCS can be used to measure trunk motion with good accuracy during kayaking.

## Supplemental Information

10.7717/peerj.15227/supp-1 Supplemental Information 1 Raw data for video camera-type kayak motion capture system and optical motion capture system.

## Additional Information and Declarations

### Data Availability

The following information was supplied regarding data availability: The raw data is available in the Supplemental File.
